# Chronic hepatitis D in Austria: high burden of cirrhosis and portal hypertension, IL28B C/C as unfavorable factor, increased systemic inflammation in decompensation

**DOI:** 10.1007/s00508-026-02700-4

**Published:** 2026-02-05

**Authors:** Michael Schwarz, Caroline Schwarz, David J. M. Bauer, Marlene Panzer, Marlene Hintersteininger, Michael Strasser, Silvia Reiter, Livia Dorn, Michael Trauner, Albert Friedrich Stättermayer, Mattias Mandorfer, Andreas Maieron, Alexander Moschen, Elmar Aigner, Heinz Zoller, Michael Gschwantler, Thomas Reiberger, Mathias Jachs

**Affiliations:** 1 Division of Gastroenterology and Hepatology, Department of Medicine III, Medical University of Vienna, Waehringer Guertel 18–20, 1090 Vienna, Austria; 2 Department of Internal Medicine 2, University Hospital of St. Pölten, St. Pölten, Austria; 3 Rare Liver Disease (RALID) Center of the European Reference Network for Rare Hepatological Diseases (ERN RARE-LIVER), Medical University Vienna, Vienna, Austria; 4 Department of Internal Medicine IV, Klinik Ottakring, Vienna, Austria; 5 Department of Internal Medicine I, Medical University of Innsbruck, Innsbruck, Austria; 6 First Department of Medicine, Paracelsus Medical University, Salzburg, Austria; 7 Department of Internal Medicine 2, Johannes Kepler University Linz, Linz, Austria; 8 Sigmund Freud University, Vienna, Austria

**Keywords:** Viral hepatitis, Liver disease, Genetic markers, ACLD

## Abstract

**Introduction:**

Chronic hepatitis D (CHD) is a severe viral hepatitis characterized by a rapid progression towards advanced chronic liver disease (ACLD). This study aimed to characterize the Austrian CHD epidemiology with respect to disease severity, systemic inflammation and genetic markers.

**Methods:**

Patients attending one of six participating hospitals from 2020 onwards were included and assessed regarding laboratory data, liver stiffness measurement (LSM) and biopsy, hepatic venous pressure gradient (HVPG) measurement, endoscopy and imaging. In a subset, single nucleotide polymorphisms (SNPs) in genes of interest (IL28B, PNPLA3, SERPINA1, NTCP) were assessed. Biomarkers of liver disease severity were compared to an age-matched cohort of treatment-eligible and treatment-naïve chronic hepatitis B (CHB) patients.

**Results:**

A total of 59 CHD patients (median age: 44.0 years, 59.3% male) were included and ACLD was found in 62.7%. Decompensated patients exhibited higher levels of biomarkers of systemic inflammation, such as C‑reactive protein (*p* = 0.061) and interleukin 6 (*p* = 0.008). The SNP IL28B C/C genotype had an unfavorable effect on disease severity (higher Model for End-Stage Liver Disease [MELD] score, HVPG and ammonia levels, lower platelet counts and albumin levels, all *p* < 0.05). The CHD patients showed a more severe liver disease compared to CHB mono-infected patients with respect to LSM, platelets, MELD and portal hypertension (all *p* < 0.01).

**Conclusion:**

This analysis of the Austrian CHD cohort before initiation of antiviral treatment showed a high burden of ACLD and portal hypertension, especially when compared to CHB patients. The IL28B C/C SNP was associated with a more severe liver disease in CHD.

**Supplementary Information:**

The online version of this article (10.1007/s00508-026-02700-4) contains supplementary material, which is available to authorized users.

## Introduction

Chronic hepatitis D (CHD) is a severe form of viral hepatitis caused by the hepatitis D virus (HDV) [1]. An HDV infection requires the concurrent infection with the hepatitis B virus (HBV) as HDV is an incomplete virion that assembles its outer coat from hepatitis B surface antigen (HBsAg) proteins [1]. Worldwide, approximately 258 million people live with chronic hepatitis B (CHB) and among these, the prevalence of anti-HDV positivity ranges from 2–13%, corresponding to an estimated 12 million people living with CHD globally [2, 3].

Compared to other forms of viral hepatitis, CHD is associated with cirrhosis and the development of hepatic decompensation, such as variceal bleeding, ascites, or hepatic encephalopathy as well as development of hepatocellular carcinoma [2, 4, 5]. Bulevirtide (BLV), a first in class sodium-taurocholate cotransporting polypeptide (NTCP) inhibitor that blocks HBV and HDV entry into hepatocytes, is the only approved treatment for CHD and recommended by international guidelines [6].

Biomarkers of systemic inflammation have been shown to increase with the progression of liver disease and are associated with liver fibrogenesis and acute hepatic decompensation [7–9]. While CHD seems to trigger a strong cytokine response, the clinical implications of successful CHD treatment require further evaluation [10].

Clinically significant portal hypertension (CSPH) is defined as an hepatic venous pressure gradient (HVPG) of ≥ 10 mm Hg and this invasively assessed parameter correlates well with clinical outcomes such as variceal bleeding or ascites [11]. Noninvasive estimation of portal hypertension includes laboratory parameters, such as platelet count, von Willebrand factor or ultrasound-derived transient elastography [11]. According to the Baveno VII consensus, CSPH can be ruled in for measurements of ≥ 25 kPa and ruled out for < 15 kPa combined with a platelet count ≥ 150 G/L [11]. These criteria have been shown to be applicable and predictive in the context of CHD [12].

Multiple genes with known effects on liver disease may also play a role in CHD. The IL28B gene encodes interferon lambda 3 (IFNL3), which induces proinflammatory genes, suppresses viral replication and genotype C/C has been associated with treatment response and spontaneous clearance in hepatitis C [13]. The NTCP is the target of BLV and single nucleotide polymorphisms (SNPs) in the NTCP were associated with a protective effect against HBV chronification and hepatocellular carcinoma (HCC) [14]. The PNPLA3 SNP G/G predisposes patients to steatotic liver disease and higher rates of steatosis among patients with CHB [15]. Alpha1-antitrypsin deficiency caused by a mutation in the *SERPINA1* gene, is a genetic chronic liver disease, but was also found to be associated with HBV chronification [16]; however, the role of these genes in the setting of CHD are unclear.

This study aimed to assess the epidemiology of people living with CHD in Austria, assess the liver disease severity before treatment initiation, explore the roles of systemic inflammation and of genes related to other liver disorders.

## Methods

### Study cohort patients

Patients with CHD from six Austrian viral hepatitis treatment centers (Medical University of Vienna, Klinik Ottakring, Medical University of Innsbruck, Medical University of Linz, Salzburg University Hospital, Sankt Pölten University Hospital), who attended their respective clinic at least once since 2020, were included.

For the comparison of compensated vs. decompensated patients, a subset of *n* = 25 no longer viremic HDV antibody positive patients were included. To investigate differences between CHD and CHB patients, a previously published treatment-naïve CHB cohort was included and 1:2 propensity score-matched to CHD patients [17]. Exclusion criteria in the control cohort were below 18 years of age, CHD, HCC and liver transplantation.

### Parameters

Demographic and clinical data, endoscopy and radiographic (signs of portal hypertension, i.e., ascites, collaterals or splenomegaly length > 12 cm, spleen size) reports were assessed. Hepatic decompensation was defined as the presence of ascites, hepatic encephalopathy and/or (history of) variceal bleeding. Laboratory-based parameters, such as Model for End-Stage Liver Disease (MELD) score, aspartate aminotransferase to platelet ratio index (APRI), Fibrosis-4 (FIB‑4) index, VITRO (Von Willebrand Factor-Antigen/Thrombocyte Ratio) and ELF (enhanced liver fibrosis) score were calculated [18, 19]. Harmful alcohol intake, defined as more than 20 g of alcohol intake per day according to local recommendations, was evaluated by the attending physician. In a subset of patients, analysis of the HDV genotype as well as analyses of SNPs in the following genes were conducted: IL28B, PNPLA3, SERPINA1, and NTCP.

Liver stiffness measurement (LSM) and steatosis assessment were performed by transient elastography and controlled attenuation parameter (CAP) assessment with Fibroscan© (Echosens, Paris, France). An LSM cut-off of ≥ 10 kPa was set according to the Baveno VII definition of advanced chronic liver disease (ACLD) [11]. As suggested by the European (i.e., the European Association for the Study of the Liver, EASL) guidance for noninvasive tests in liver disease, liver steatosis was assumed in patients with controlled attenuation parameter (CAP) values above 275 dB/m [18]. For the noninvasive assessment of portal hypertension, the Baveno VII criteria were applied: rule-out CSPH: LSM ≤ 15 kPa and platelet count ≥ 150G/L; rule-in CSPH: LSM ≥ 25 kPa [11, 12]. ACLD was defined as a compound parameter consisting of LSM ≥ 10 kPa, liver biopsy of F3/F4, and/or HVPG ≥ 6 mm Hg.

Quantitative HDV RNA PCRs were performed the Department of Laboratory Medicine at the Medical University of Vienna using an in-house assay which had a lower limit of detection of 100 copies/mL. The Medical University of Innsbruck used the RoboGene HDV Quantification Kit 2.0 (Roboscreen Diagnostics, Leipzig, Germany) with a lower limit of quantification of 8 IU/ml and a validated conversion factor of 37 was applied [20].

### Statistics

For data curation, Microsoft Excel was used (Office 2019, Microsoft, Redmond, WA, USA). For statistical analysis, Rstudio (Build 764, Posit Software, Boston, MA, USA) was used. For graphical display, GraphPad Prism (version 9, GraphPad, Boston, MA, USA) and RStudio were used. Continuous variables are presented as median (and interquartile range, IQR, Q1–Q3) or as frequencies (*n*, and %). For statistical comparison χ^2^-test, Wilcoxon signed-rank test, Mann-Whitney U test or Kruskal-Wallis test were used. Box plots include 10–90th percentile whiskers and outliers.

### Ethics

The study was conducted in concordance with the principles of the Declaration of Helsinki and approved by the ethics committee of the Medical University of Vienna (ethics committee numbers: 1515/2020 and 2139/2021). Written informed consent was obtained from CHD patients treated at the Vienna General Hospital and Klinik Ottakring (*n* = 49, 83.1%), while written informed consent was waived by the Institutional Review Board (IRB) for the retrospective inclusion of the remaining patients.

## Results

### Patient characteristics and epidemiology

**Fig. 1:**
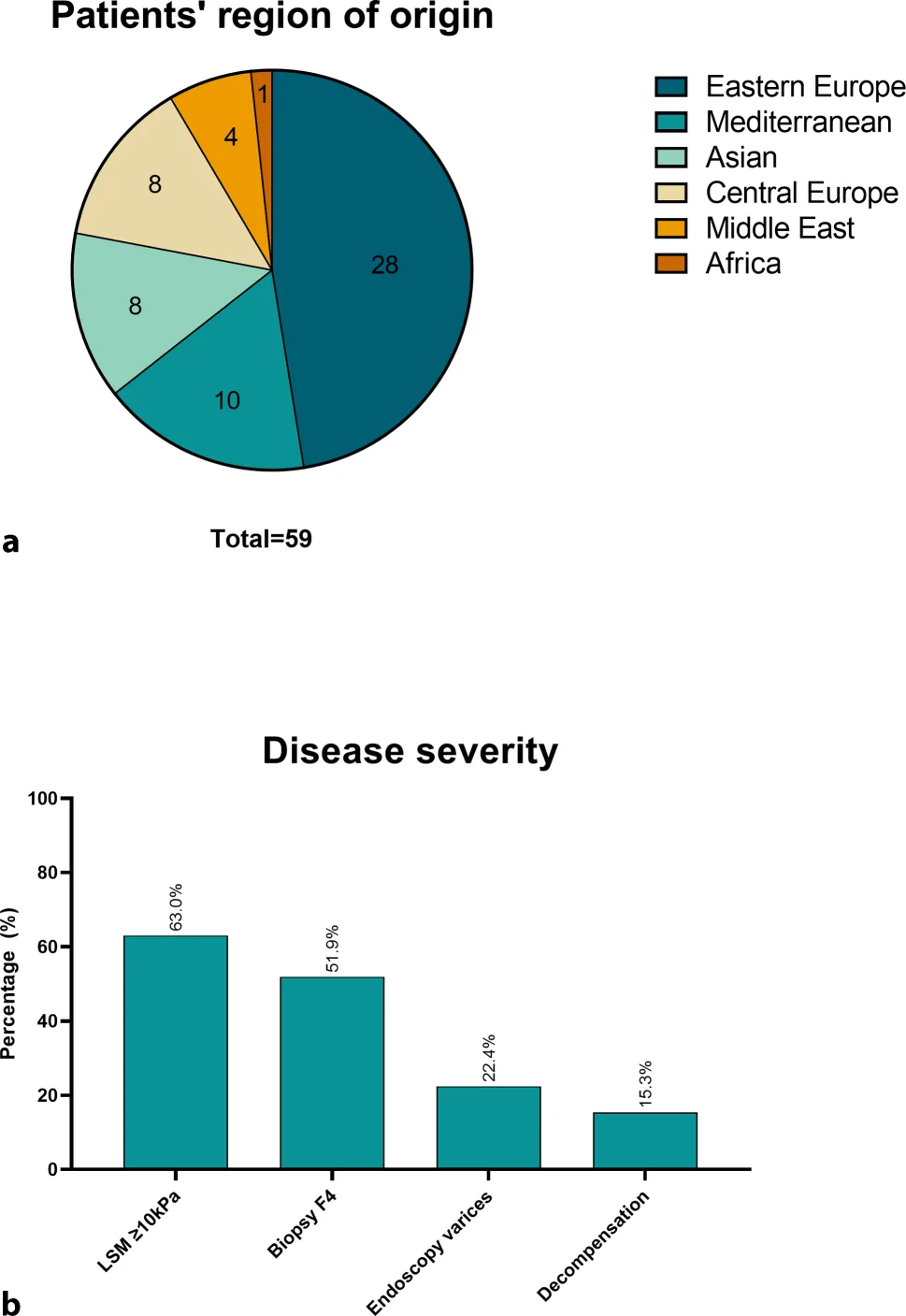
Patient origin and liver disease severity. **a** The majority of patients were originally from Eastern Europe, the Mediterranean Region and Asia. Most common nationalities were Romania and Mongolia. **b** Regarding liver disease severity, a high burden of ACLD, cirrhosis in biopsy and portal hypertension was found among patients with chronic hepatitis D.

In total, *n* = 59 HDV viremic patients were included. The majority of patients were treated at the Vienna General Hospital (*n* = 36 patients, 61.0%) or at Klinik Ottakring (*n* = 13 patients, 22.0%). Other patients were recruited at the following centers across Austria: Medical University of Innsbruck *n* = 5 (8.5%), Johannes Kepler University Linz *n* = 2 (3.4%), Paracelsus Medical University Salzburg *n* = 2 (3.4%), and University Hospital Sankt Pölten *n* = 1 (1.7%). The median age was 44.0 years (IQR 37–52.5 years) and 59.3% of patients were male. Eastern Europe was the most common region of origin (*n* = 28, 47.5%), followed by the Mediterranean Region (*n* = 10, 16.9%), Asia and Central Europe (both *n* = 8, 13.6%). Most common countries of origin were Romania (*n* = 16, 27.1%), Austria (*n* = 7, 11.9%), Turkey (*n* = 6, 10.2%), Mongolia and Moldova (both *n* = 5, 8.5%). All patients were naïve to HDV-specific antiviral treatment with BLV and *n* = 53 (89.8%) received nucleo(t)ide analogue (NA) treatment for CHB at the time of characterization. See Table 1 and Fig. 1.

**Table 1:** Baseline characterization of CHD patients with respect to epidemiology, comorbidities, liver disease, portal hypertension and single nucleotide polymorphisms in genes related to liver disease.

Patient characteristics (*n* = 59)	Median (Q1; Q3) or *n* (%)
**Age (years)**	44.0 (37.0; 52.5)
**Sex**	
Female	24 (40.7%)
Male	35 (59.3%)
**Region of origin**	
Eastern Europe	28 (47.5%)
Mediterranean Region	10 (16.9)
Asia	8 (13.6%)
Central Europe	8 (13.6%)
Africa	1 (1.7%)
Middle East	4 (6.8%)
**Body mass index**	23.8 (21.8; 28.7)
**Diabetes mellitus type 2**	
None	55 (93.2%)
Non-insulin-dependent	3 (5.1%)
Insulin-dependent	1 (1.7%)
**Daily alcohol intake**	6 (10.2%)
**HIV co-infection**	6 (10.2%)
**Anti-HCV antibodies**	8 (13.6%, none viremic)
**HDV genotype**	
GT 1	28/28 (100%)
**HDV RNA PCR in copies/mL**	1.3E + 05 (8.3E + 03; 4.3E + 05)
**ACLD**	37 patients (62.7%)
**Biopsy stage (*n*** **=** **27, 45.8%)**	
F0/F1	3 (11.1%)
F2	4 (22.2%)
F3	6 (22.2%)
F4	14 (51.9%)
**LSM in kPa (*n*** **=** **54, 91.5%)**	13.1 (8.6; 18.9)
≥ 10 kPa	34 patients (63.0%)
> 15 kPa	19 patients (35.2%)
**CAP in dB/m (*n*** **=** **31, 52.5%)**	203 (182; 259)
> 275 dB/m	6 (19.4%)
**MELD score in points (*n*** **=** **55, 93.2%)**	8.00 (7.0; 10.0)
≥ 15	2 patients (3.7%)
**APRI score (*n*** **=** **57, 96.6%)**	0.83 (0.54; 1.70)
**FIB‑4 score (*n*** **=** **57, 96.6%)**	2.40 (1.27; 3.98)
**VITRO score (*n*** **=** **32, 54.2%)**	1.82 (0.861; 3.510)
**ELF test score (*n*** **=** **22, 37.3%)**	10.5 (9.5; 11.6)
**HVPG in mm** **Hg (*n*** **=** **24, 40.7%)**	10.5 (6.0; 17.3)
≥ 10 mm Hg	15 patients (62.5% of 24)
**Ascites**	7 (11.9%)
**Hepatic encephalopathy**	9 (15.3%)
**Ammonia in µmol/L**	27.0 (21.5; 39.1)
**Esophageal varices (*n*** **=** **49, 83.1%)**	
None	38 (77.6%)
Small	6 (12.2%)
Large	5 (10.2%)
**Imaging for portal hypertension (*n*** **=** **51, 86.4%)**	
No signs of portal hypertension	41 (80.4%)
Splenomegaly, collaterals, ascites	10 (19.6%)
Median spleen size in cm	12.5 (10.8; 15.9)
**Single nucleotide polymorphisms**	
*IL28B (n* *=* *36)*	
C/C	17 (47.2%)
T/T	3 (8.3%)
C/T	16 (44.4%)
*PNPLA3 (n* *=* *39)*	
G/G	3 (7.7%)
C/G	22 (56.4%)
C/C	14 (35.9%)
*SERPINA1*	
rs17580 WT	36/36 (100%)
rs28929474 WT	31/31 (100%)
*NTCP (n* *=* *32)*	
C/C	31 (100%)

*ACLD* advanced chronic liver disease, *APRI* aspartate aminotransferase to platelet ratio index, *CAP* Controlled Attenuation Parameter, *ELF* Enhanced Liver Fibrosis test, *FIB4* Fibrosis-4 index, *HDV* hepatitis D virus, *LSM* liver stiffness measurement, *HIV* human immunodeficiency virus, *MELD* Model for End-stage Liver Disease, *NTCP* sodium-taurocholate cotransporting polypeptide, *PCR* polymerase chain reaction, *PNPLA3* Patatin-like phospholipase domain-containing protein 3, *RNA* ribonucleic acid, *VITRO* Von Willebrand Factor-Antigen/Thrombocyte Ratio, *WT* wild type

### Noninvasive and invasive assessment of liver disease severity in CHD patients

An ALCD was found in *n* = 37 (62.7%) of patients and LSM was available in 54 patients (91.5%). Of these, *n* = 34 (63.0%) had ≥ 10 kPa and *n* = 19 (35.2%) above 15 kPa. Steatotic liver disease was present in 19.4% (*n* = 6 of 31 patients). Biopsy data were available in *n* = 27 (45.8%) patients and *n* = 14 (51.9%) had F4 cirrhosis. The median Fibrosis-4 (FIB4) index was 2.40 (IQR 1.27–3.98) with *n* = 26 (45.6% of 57) of patients above the threshold of > 2.67 indicative of advanced fibrosis [21]. Of these, *n* = 22 had LSM and *n* = 19 (86.4%) had ≥ 10 kPa. The median APRI score was 0.828 (IQR 0.54–1.70) and *n* = 18 (31.6% of 57) patients were above the fibrosis cut-off of 1.5. Compared with available LSM data, 12 (85.7%) of 14 of these patients had a LSM of ≥ 10 kPa. The median ELF test score was 10.5 (IQR 9.45–11.6) and *n* = 13 (59.1% of 22) had a score above 9.8 points, the discriminative cut-off for the identification of fibrosis. Of these, *n* = 12 (92.3%) had a LSM ≥ 10 kPa. The median VITRO score was 1.82 (IQR 0.86–3.51) and *n* = 13 (40.6% of 32) patients had values ≥ 2.5 indicative of clinically significant portal hypertension (CSPH) [19, 22]. Of these patients 11 underwent HVPG measurement and *n* = 9 (81.8%) had CSPH.

An HVPG assessment was available in *n* = 24 (40.7%) patients and the median HVPG was 10.5 (IQR 6.0–17.3) mm Hg. In total, *n* = 15 (62.5% of *n* = 24) patients had an HVPG of ≥ 10 mm Hg and thus, CSPH. At the time of characterization, *n* = 7 (11.9%) patients were decompensated with ascites and *n* = 9 (15.3%) had hepatic encephalopathy. The median ammonia level (NH3) was within the normal range with a median value of 27.0 (21.5–39.1) µmol/L (normal range 11–51 µmol/L). See Table 1 and Fig. 1.

When stratifying according to Baveno VII criteria, increasing levels of bile acids (rule-out CSPH bile acids 4.70, IQR 3.6–14.4 vs. rule-in 13.6, 8.5–24.8 µmol/L, *p* = 0.118) and markers of systemic inflammation, such as IL6 (rule-out 2.78, IQR 1.81–3.44 vs. rule-in 4.53, IQR 4.49–13.4pg/mL, *p* = 0.074) and PCT (rule-out 0.08, IQR 0.03–0.10 vs. rule-in 0.14, IQR 0.12–0.15ng/dL, *p* = 0.149) were observed in the CSPH rule-in group. See Supplementary Table 1 and Supplementary Fig. 1.

### Systemic inflammation and markers of liver disease in decompensated patients

**Fig. 2:**
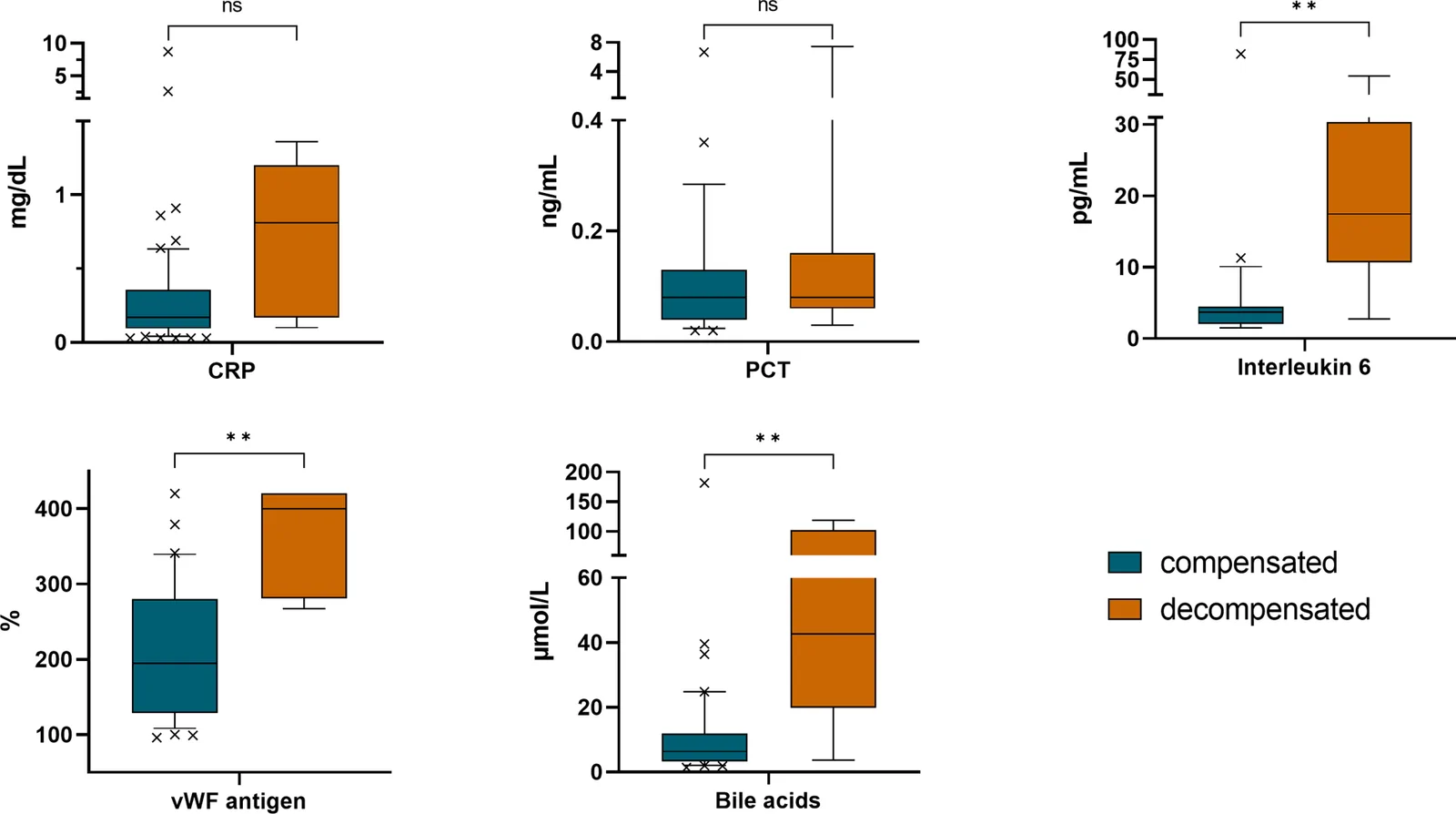
Comparison of markers of systemic inflammation in compensated and decompensated patients. When comparing compensated (*n* = 72) and decompensated (*n* = 12) anti-HDV positive patients before treatment initiation (*n* = 59 viremic patients), decompensated patients had higher levels of markers of systemic inflammation like C‑reactive protein and interleukin 6. Von Willebrand factor (vWF, *p* = 0.001), as an indicator of portal hypertension/endothelial dysfunction, and bile acids were also significantly elevated in decompensated patients. *p < 0.05, **p < 0.01. *CRP* C-reactive protein, *PCT* procalcitonin, *vWF* von Willebrand Factor

For a sub-analysis, *n* = 25 anti-HDV positive but no longer HDV RNA positive patients were included. This resulted in *n* = 84 patients in total, *n* = 72 (85.7%) with compensated and *n* = 12 (14.3%) with decompensated ACLD. Decompensated patients showed higher levels of markers of systemic inflammation, such as CRP (0.81, IQR 0.17–1.20 vs. 0.17, IQR 0.10–0.33 mg/dL, *p* = 0.061) and interleukin 6 (IL6) (17.5, IQR 13.8–21.5 vs. 3.67, IQR 2.04–4.45 pg/dL, *p* = 0.008). Decompensated patients had higher levels of bile acids (42.7, IQR 25.6–87.8 vs. 6.35, IQR 3.32–11.4 µmol/L, *p* = 0.006). There was no significant difference in levels of CRP, IL6, PCT, or bile acids between HDV RNA positive and negative patients with anti-HDV antibodies. Noninvasive assessments of liver fibrosis and/or portal hypertension, such as FIB‑4, APRI, and VITRO were elevated in decompensated patients (all *p* < 0.05). See Fig. 2 and Supplementary Tables 2 and 3.

When excluding HDV RNA negative, anti-HDV antibody positive patients (*n* = 22 compensated, *n* = 3 decompensated) and only comparing HDV viremic patients, higher levels of CRP and IL6 were still observed among patients with decompensated liver disease; however, these results did not attain statistical significance (CRP 0.29 [IQR 0.11–0.42] vs. 0.48 [IQR 0.19–1.14] mg/dL, *p* = 0.191; IL6 3.71 [IQR 2.40–4.47] vs. 15.3 [9.04–18.8] pg/dL, *p* = 0.144). See Supplementary Fig. 2 and Supplementary Table 4.

### Genetic polymorphisms in HDV

The SNPs in PNPLA3, IL28B, SERPINA1, and NTCP were assessed in a subset of patients. Data for IL28B polymorphism were available for *n* = 36 (61.9%) patients, whereof *n* = 17 (47.2%) carried genotype C/C, *n* = 3 (8.3%) T/T, and *n* = 16 (44.4%) C/T. Data on PNPLA3 polymorphisms were available in *n* = 39 (66.1%) of *n* = 59 viremic patients (G/G *n* = 3 [7.7%], C/C *n* = 14 [35.9%], C/G *n* = 22 [56.4%]). No SNPs were found in NTCP or SERPINA1. See Table 1.

**Fig. 3:**
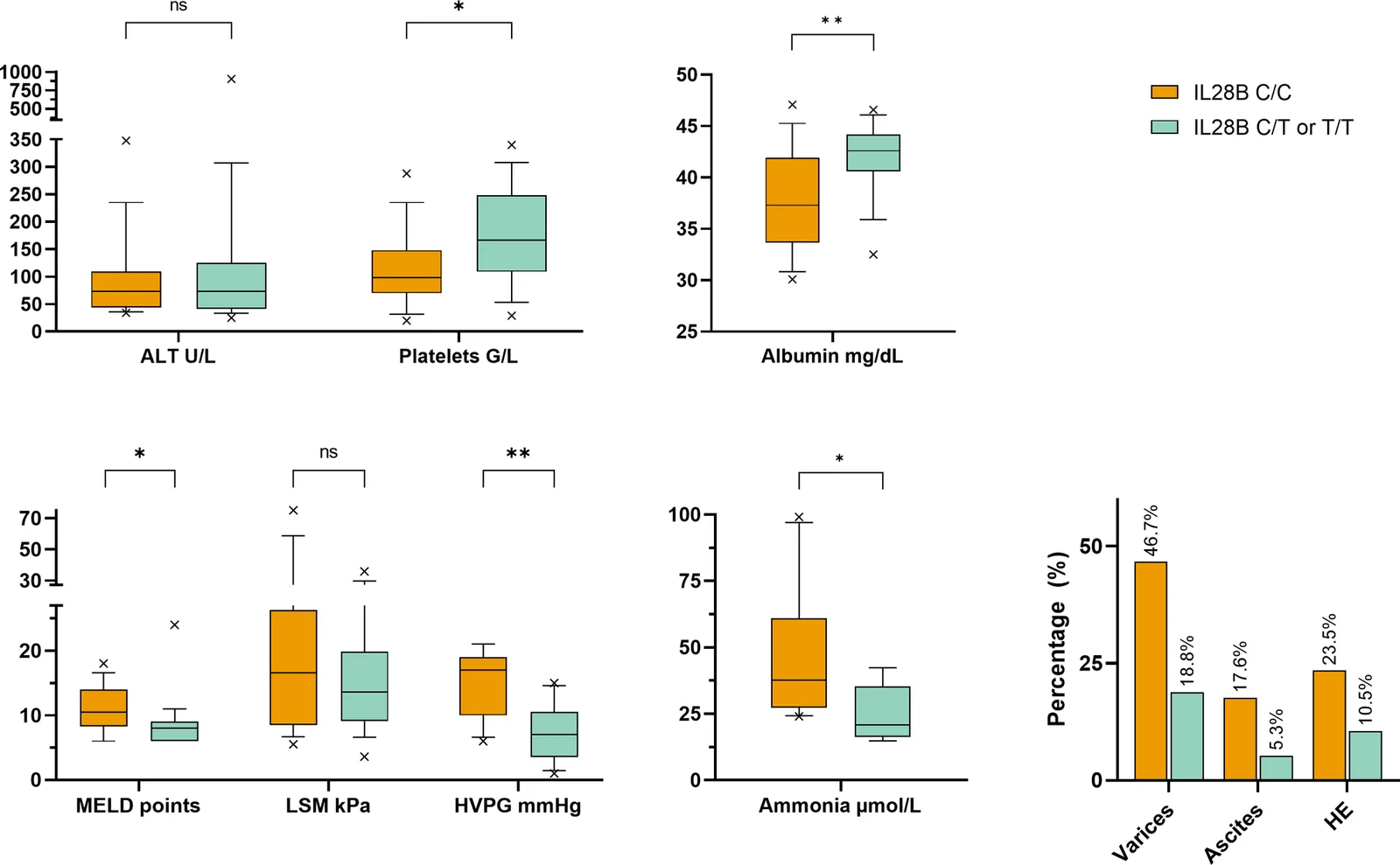
Effects of IL28B single nucleotide polymorphisms on liver disease severity. The CHD patients with genotype IL28B C/C exhibited a more severe liver disease in invasive and noninvasive assessments and also more frequently had clinical complications. *ALT* alanine aminotransferase, *HE* hepatic encephalopathy, *HVPG* hepatic venous pressure gradient, *LSM* liver stiffness measurement, *MELD* Model for End-Stage Liver Disease

Regarding IL28B SNPs, the cohort was stratified into patients with the “favorable” C/C genotype (*n* = 17) and the genotypes C/T or T/T (*n* = 19). Carriers of the C/C SNP had lower platelets (98 [IQR 77–134] vs. 166 [110–241] G/L, *p* = 0.017), lower albumin (37.3 [IQR 33.7–39.9] vs. 42.6 [IQR 41.0–44.2] mg/dL, *p* = 0.006), higher INR (1.3 [1.2–1.6] vs. 1.1 [1.0–1.2], *p* = 0.003), higher ammonia levels (37.7 [IQR 28.9–53.5] vs. 20.9 [16.4–29.1] µmol/L, *p* = 0.013), a higher MELD score (10.5 [IQR 8.75–12.2] vs. 8.0 [6.0–9.0] points, *p* = 0.019), and an higher HVPG (17 [IQR 10.5–19.0] vs. 6.5 [4.0–10.0] mm Hg, *p* = 0.004). Concordantly, C/C patients had higher LSM (16.6 [IQR 9.5–25.4] vs. 13.6 [IQR 9.7–19.2], n. s.) and of *n* = 11 performed biopsies in C/C patients, all were F3/F4. Data on IL28B SNP were unfortunately only available for a single patient in the non-viremic (HDV RNA negative, anti-HDV antibody positive) subgroup. This patient had genotype T/T. For PNPLA3 SNPs no relevant effect on viral load or disease severity was observed. See Fig. 3 and Supplementary Tables 5 and 6.

### Liver disease severity in CHD vs. CHB

To compare disease severity between patients with untreated CHD and patients with untreated CHB, a control cohort of *n* = 118 age-matched HBV mono-infected and nucleos(t)ide analogue (NA) treatment-eligible and treatment-naïve patients was included. See Table 2 for patient details.

**Table 2:** Comparison between patients with active HDV viremia (*n* = 59) and patients with chronic hepatitis B mono-infection eligible for nucleoside analogue (NA) treatment initiation (*n* = 118)

Parameter	HDV RNA+ (*n* = 59)	HBV NA (*n* = 118)	Pval
*Age in years*	44.0 (37.0; 52.5)	44.0 (36.6; 52.8)	0.998
*Sex*			0.125
Male	35 (59.3%)	85 (72.0%)	–
Female	24 (40.7%)	33 (28.0%)	–
*BMI*	23.8 (21.8; 28.7)	25.0 (23.8; 28.0)	0.213
*Daily alcohol intake*			1.000
No	53 (89.8%)	42 (89.4%)	–
Yes	6 (10.2%)	5 (10.6%)	–
*HIV coinfection*			0.238
No	53 (89.8%)	38 (97.4%)	–
Yes	6 (10.2%)	1 (2.56%)	–
*LSM in kPa*	13.1 (8.55; 18.9)	8.80 (6.48; 12.8)	*0.002*
*LSM stages:*			
kPa ≥ 10	34 (63.0% of 54)	32 (41.0% of 78)	*0.021*
kPa > 15	19 (35.2%)	12 (15.4%)	*0.015*
*Ascites and/or HE*	*9 (15.3%)*	*4 (3.4%)*	*0.011*
*Liver disease severity*			
APRI score	0.83 (0.54; 1.7)	0.39 (0.24; 0.79)	*<* *0.001*
FIB4 score	*2.40 (1.27; 3.98)*	*1.24 (0.67; 1.95)*	*<* *0.001*
MELD UNOS	*8.00 (7.00; 10.0)*	*7.00 (6.00; 9.00)*	*<* *0.001*
*Baveno VII criteria*			
Rule-out CSPH	21 (38.9%)	55 (70.5%)	**–**
Gray zone	25 (46.3%)	17 (21.8%)	*0.001*
Rule-in CSPH	8 (14.8%)	6 (7.69%)	**–**
*Laboratory parameters*			
HBV DNA U/mL	1.00E + 01 (0; 2.35E + 01)	5.72E + 04 (1.04E + 04; 3.16E + 06)	*<* *0.001*
HBV DNA < LLD	41 (74.5%) of 55	118 (100%) of 118	*<* *0.001*
HBs antigen IU/mL	7514 (2090; 14,079)	4000 (1892; 12,503)	0.344
Platelets in G/L	134 (91.5; 204)	208 (162; 245)	*<* *0.001*
*INR*	*1.20 (1.00; 1.30)*	*1.09 (1.00; 1.13)*	*<* *0.001*
*AST in U/L*	*51.0 (39.0; 68.0)*	*38.0 (27.0; 59.8)*	*0.001*
ALT in U/L	66.0 (42.0; 107)	66.5 (36.0; 93.5)	0.432
Bilirubin in mg/dL	0.66 (0.50; 1.10)	0.58 (0.39; 0.92)	0.066
Albumin in mg/dL	42.6 (37.3; 44.4)	43.5 (40.7; 45.5)	0.053
Alk. phosphatase U/L	83.0 (71.0; 120)	68.5 (57.0; 94.8)	*<* *0.001*
Creatinine in mg/dL	0.79 (0.65; 0.86)	0.81 (0.71; 0.92)	0.090
Sodium in mmol/L	139 (137; 140)	140 (139; 142)	*0.010*

CHB patients were eligible for antiviral treatment, but still treatment naïve, had no detectable HDV RNA, and were propensity score matched to CHD patients by age. In general, CHD patients had a more advanced liver disease at baseline, shown by higher MELD, APRI and FIB4 as well as lower platelet counts and albumin. Missing data not shown. *ALT* alanine aminotransferase, *APRI* aspartate aminotransferase to platelet ratio index, *AST* aspartate aminotransferase, *BMI* Body Mass Index, *CSPH* clinically significant portal hypertension, *DNA* deoxyribonucleic acid, *FIB4* Fibrosis-4 Index, *HBV* hepatitis B virus, *HDV* hepatitis D virus, *HIV* human immunodeficiency virus, *INR* International Normalized Ratio, *LLD* lower limit of detection, *MELD* Model for End-Stage Liver Disease, *RNA* ribonucleic acid, *UNOS* United Network for Organ Sharing

The CHD patients had higher LSM compared to CHB patients (CHD vs. CHB: 13.1 [8.6–18.9] vs. 8.8 [IQR 6.5–12.8] kPa, *p* = 0.002), with ACLD on transient elastography commonly found. Noninvasive assessments of liver fibrosis, such as the APRI and FIB4 scores, were significantly higher among CHD patients (both *p* < 0.001). Biopsies were available in *n* = 27 CHD patients and *n* = 9 CHB patients. Cirrhosis was more commonly found in CHD patients (*n* = 14 [51.9%] of 27 vs. 1 [11.1%] of 9, *p* = 0.051).

**Fig. 4:**
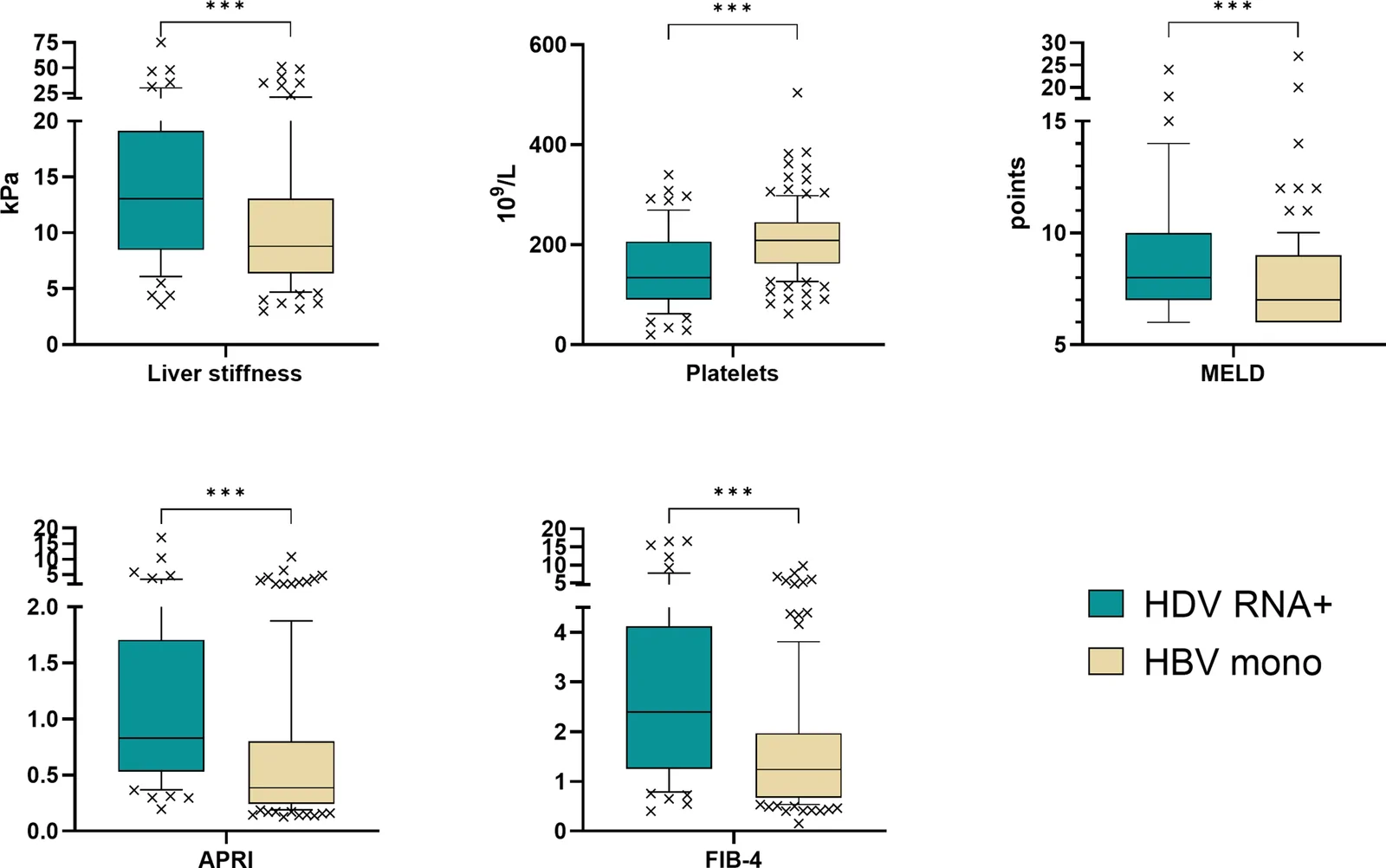
Comparison of markers of liver fibrosis and portal hypertension between treatment eligible hepatitis B virus (HBV) mono-infected patients and CHD patients. CHD patients showed significantly higher liver fibrosis scores (APRI, FIB-4) and higher MELD, and exhibited non-invasive indicators of portal hypertension such as higher liver stiffness measurements (LSM) and lower platelets (all *p* < 0.001). *APRI* aspartate aminotransferase to platelet ratio index, *FIB4* Fibrosis-4 Index, *HBV* hepatitis B virus, *HDV* hepatitis D virus, *MELD* Model for End-Stage Liver Disease

The CHD patients commonly had an HBV DNA below the limit of detection (i.e. 20 IU/mL, *n* = 41 [74.5%] of 55 with available data) and lower HBV DNA levels in general (1.00E + 01 [IQR 0–2.35E + 01] vs. 5.72E + 04 [IQR 1.04E + 04–3.16E + 06], *p* < 0.001), while HBs antigen levels did not differ significantly (*p* = 0.582). See Fig. 4 and Table 2.

### Portal hypertension in CHD vs. CHB patients

The CHD patients exhibited significantly lower platelet counts compared to CHB patients (134 G/L [IQR 91.5–204] vs. 208 G/L [IQR 162–245], *p* < 0.001). Decompensation was more prevalent in the CHD cohort compared to the CHB cohort (CHD 9 of 59 [15.3%] vs. CHB 4 of 118 [3.4%], *p* = 0.010). See Table 2.

**Fig. 5:**
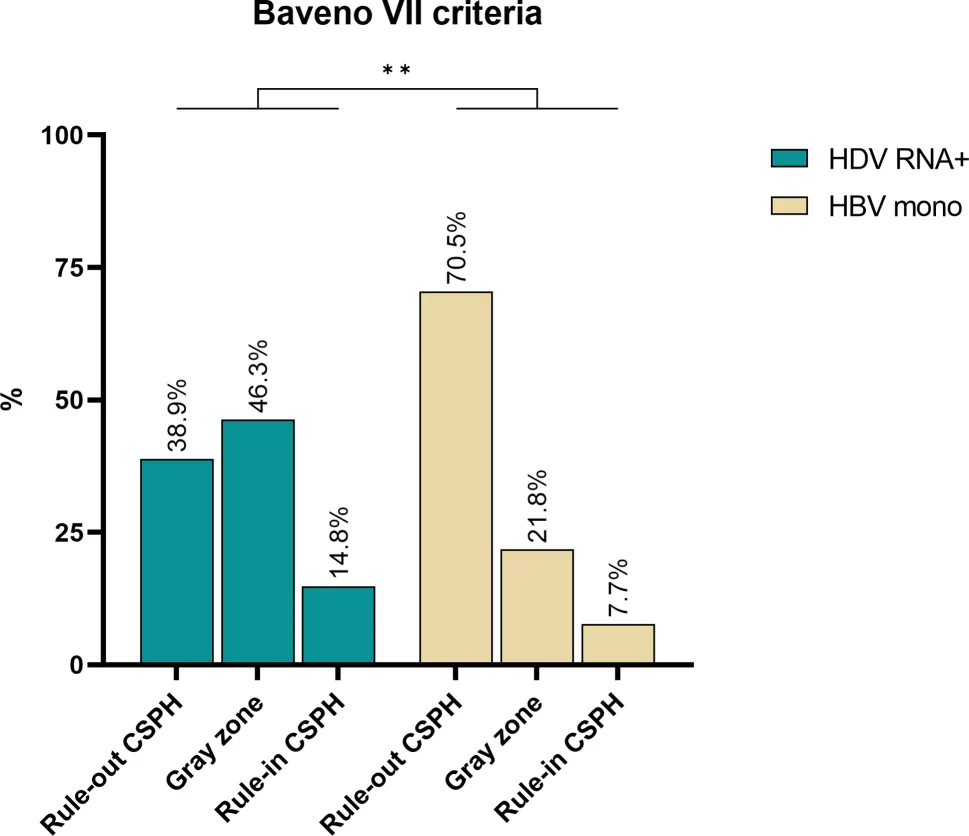
Application of Baveno VII criteria for the noninvasive assessment of clinically significant portal hypertension (CSPH). Patients with chronic hepatitis D and the age-matched nucleoside analogue-naïve HBV mono-infected cohort were stratified according to the Baveno VII criteria. CSPH was more frequently ruled out in the CHB cohort (*p* = 0.001). *CSPH* clinically significant portal hypertension, *HBV* hepatitis B virus, *HDV* hepatitis D virus

When stratifying viremic CHD patients (*n* = 59) and age-matched CHB patients (*n* = 118) according to the Baveno VII criteria, CSPH was more frequently ruled-out among CHB patients (70.5% in CHB vs. 38.9% in CHD, *p* = 0.001). CHD patients showed a relatively large portion of “gray zone” patients (25 of 54 classifiable patients, 46.3%), 4 (16%) of which had varices on endoscopy. Among CHB patients, *n* = 34 (32.1%) of 106 classifiable patients were within the “gray zone”, 3 (9.4%) of which also had esophageal varices. See Supplementary Table 1, and Fig. 5.

## Discussion

Through this nationwide collaboration of six Austrian viral hepatitis centers, a well-characterized cohort of CHD patients before treatment initiation was created. Common regions of origin in our cohort were Eastern Europe, the Mediterranean region, or Asia. Eastern European patients were predominantly from Romania, Mediterranean patients from Turkey, and Asian patients were commonly of Mongolian descent. All of these countries are known for a high prevalence of anti-HDV antibodies [3].

The cohort of was defined by a high burden of ACLD and portal hypertension. By applying the Baveno VII LSM cut-off of 10 kPa, 63.0% of patients in our cohort had values suggestive of ACLD. Regarding LSM in CHD, different cut-off values ranging from 10–15.2 kPa have been proposed before [23, 24]. Applying a cut-off of 15 kPa to our cohort reduced the ACLD percentage to 35.2%, which might be underrepresentative. Laboratory-based fibrosis tests FIB‑4, APRI, and ELF performed similar to LSM values ≥ 10 kPa (FIB4 86.4%, APRI 85.7%, ELF 92.3% accuracy).

In hepatitis C, IL28B SNP C/C is associated with spontaneous clearance of HCV infection and response to PEGIFN-based treatment regimens [13] but data regarding IL28B’s role in CHD seem conflicting or tend towards no effect on viral clearance and liver disease severity [25–27]; however, in our *n* = 36 CHD patients with IL28B SNP data, we observed that the “favorable” genotype C/C was associated with a more severe liver disease and portal hypertension. Potentially and similarly to the effect in chronic hepatitis C, IL28B C/C may cause a proinflammatory phenotype leading to pronounced necroinflammation and fibrosis [28]; however, while this may benefit viral clearance in CHC, the effect might not translate to CHD, but instead, the ongoing inflammation without viral clearance might drive disease progression.

When comparing untreated CHD with untreated CHB, a more severe liver disease with higher APRI, FIB4, and LSM, higher MELD score was observed for CHD patients, as expected. A recent study from Germany, which assessed the clinical course of CHB and CHD patients, found that while CHD progresses to ACLD in a more rapid fashion, once ACLD is established this seems to be the driving force behind further complications of liver disease [4].

Systemic inflammation increases with liver disease progression and portal hypertension and is associated with worse prognosis [8, 9]. In our CHD patients with ruled-in CSPH, humoral markers of portal hypertension and systemic inflammation changed accordingly. Although the changes in systemic inflammation were not statistically significant, they tended in the expected direction. When comparing decompensated and compensated patients, again increased levels of systemic inflammation were observed. Potentially, disruption of these inflammatory processes via antiviral treatment with BLV may improve liver function and enable disease regression. As of now, BLV is only approved for patients with compensated liver disease but may be safe in certain patients with decompensated liver disease [29]. Furthermore, among CHD CSPH rule-in patients, serum levels of bile acids were elevated. This may be expected in ACLD but might also be interesting for future research as BLV’s target is the bile acid uptake system NTCP [6].

With *n* = 59 CHD patients, the sample size was relatively small, especially when dividing the patients into further subgroups; however, the patients in our cohort are well characterized and the findings were in themselves coherent. To address the small cohort size, we included *n* = 25 anti-HDV antibody positive, HDV RNA negative patients (*n* = 22 [88.0%] with compensated liver disease) for a subanalysis and found elevated markers of systemic inflammation in patients with decompensated liver disease. When repeating the analysis with the original dataset (*n* = 59), higher levels of CRP and IL6 were observed in decompensated patients but these results did not attain statistical significance. Subdivision of our cohort resulted in relatively small subgroups, limiting statistical interpretation and, regarding humoral markers of systemic inflammation like CRP, potentially facilitating distortion through causes of inflammation other than ACLD; however, as the observed tendencies remained robust throughout, further analysis in larger cohorts may be warranted.

In summary, the presented data of CHD patients from six different Austrian centers provides a relevant estimation of the overall epidemiology of hepatitis delta in our country, capturing almost all of the viremic patients who are still in follow-up in Austria [30]. The Austrian CHD cohort demonstrated a high burden of ACLD and portal hypertension, especially compared to HBV mono-infected patients. Decompensated liver disease was associated with elevated markers of systemic inflammation. The SNP IL28B C/C was associated with more severe liver disease and portal hypertension, suggesting a potentially complex role for this genetic variant in CHD disease progression.

## Supplementary Information

Supplementary Table 1: Application of the Baveno VII criteria for non-invasive diagnosis of portal hypertension in the CHD and CHB cohort

Supplementary Table 2: Comparison of compensated and decompensated patients at the time of characterization

Supplementary Table 3: Comparison of HDV RNA positive and HDV RNA negative patients with anti-HDV antibodies.

Supplementary Table 4: Comparing patients with compensated and decompensated liver disease with respect to HDV RNA positivity.

Supplementary Table 5: CHD patients’ characteristics stratified by IL28B status.

Supplementary Table 6: Characteristics of CHD patients stratified by PNPLA3 status

Supplementary Fig. 1: Markers of systemic inflammation and portal hypertension in CHD patients stratified according to Baveno VII assessment of portal hypertension

Supplementary Fig. 2: Comparison of markers of systemic inflammation in HDV RNA positive patients with compensated and decompensated chronic liver disease

## Data Availability

Anonymized data are available from the authors on reasonable request.
