# Supplementary material for: Chronic hepatitis D in Austria: high burden of cirrhosis and portal hypertension, IL28B C/C as unfavorable factor, increased systemic inflammation in decompensation
Source: Wien Klin Wochenschr. 2026 Feb 5;138(15-16):483–94. doi: 10.1007/s00508-026-02700-4 (PMC13493468; doi:10.1007/s00508-026-02700-4)
Supplement: Supplementary file 1 [file 508_2026_2700_MOESM1_ESM.docx]

| **Baveno VII:** | **Rule-out CSPH** | **Gray zone** | **Rule-in CSPH** | **pval** |
| --- | --- | --- | --- | --- |
| ***HBV NA cohort*** | n=55 | n=17 | n=6 |  |
| Age in years | 43.2 (36.4; 52.0) | 38.5 (37.1; 51.8) | 48.0 (43.9; 51.6) | 0.526 |
| BMI | 24.9 (23.8; 27.1) | 24.6 (20.5; 27.4) | 31.7 (30.4; 34.3) | **0.014** |
| **Laboratoy parameters** | |  |  |  |
| HBV DNA in IU/mL | 4.95E+04 (1.68E+04; 1.52E+06) | 2.39E+05 (1.96E+03; 8.35E+06) | 1.25E+05 (1.13E+02; 2.63E+06) | 0.575 |
| Platelets in G/L | 209 (178; 241) | 130 (116; 149) | 197 (140; 259) | **<0.001** |
| AST in U/L | 36.0 (27.0; 43.5) | 78.0 (48.0; 138) | 98.5 (48.0; 192) | **<0.001** |
| ALT in U/L | 73.0 (35.5; 81.0) | 69.0 (48.0; 145) | 96.0 (64.2; 100) | 0.127 |
| **Liver disease severity** | |  |  |  |
| LSM in kPa | 7.80 (5.85; 10.2) | 12.9 (9.50; 16.6) | 38.5 (35.3; 47.0) | **<0.001** |
| APRI | 0.36 (0.25; 0.51) | 1.39 (0.82; 2.01) | 1.65 (0.70; 1.82) | **<0.001** |
| FIB-4 | 1.03 (0.64; 1.30) | 2.45 (1.87; 3.41) | 3.18 (1.56; 4.03) | **<0.001** |
| MELD UNOS | 7.00 (6.00; 8.00) | 8.00 (7.00; 10.0) | 10.0 (8.50; 10.0) | **0.018** |
|  |  |  |  |  |
| ***HDV RNA+ cohort*** | *n=21* | *n=25* | *n=8* |  |
| Age in years | 38.0 (35.0; 50.0) | 45.0 (38.0; 56.0) | 52.5 (45.8; 61.5) | **0.048** |
| BMI | 23.7 (22.1; 28.2) | 24.4 (21.7; 30.3) | 25.9 (22.7; 28.9) | 0.794 |
| **Laboratory parameters** |  |  |  |  |
| HDV RNA | 2.4E+05 (1.4E+04; 5.2E+05) | 1.2E+04 (6,2E+03; 1.6E+05) | 3.2E+05 (7.1E+04; 5.3E+05) | 0.051 |
| HBV DNA | 1.00E+01 (0; 6.00E+01) | 0 (0; 2.85E+01) | 0 (0; 1.00E+01) | 0.083 |
| Platelets in G/L | 208 (177; 251) | 109 (85.0; 127) | 98.0 (81.5; 128) | **<0.001** |
| AST in U/L | 47.0 (39.0; 55.0) | 51.0 (35.5; 69.5) | 56.0 (52.2; 74.8) | 0.334 |
| ALT in U/L | 73.0 (42.0; 102) | 56.0 (44.0; 115) | 69.5 (60.5; 102) | 0.751 |
| INR | 1.06 (1.0; 1.20) | 1.2 (1.06; 1.30) | 1.30 (1.15; 1.65) | **0.018** |
| CRP in mg/dL | 0.29 (0.09; 0.51) | 0.29 (0.13; 0.40) | 0.25 (0.15; 0.31) | 0.990 |
| IL6 in pg/mL | 2.78 (1.81; 3.44) | 3.79 (2.73; 4.57) | 4.53 (4.49; 13.4) | 0.074 |
| PCT in ng/mL | 0.08 (0.03; 0.10) | 0.08 (0.05; 0.14) | 0.14 (0.12; 0.15) | 0.149 |
| vWF antigen perc. | 177 (138; 237) | 245 (178; 302) | 326 (296; 330) | **0.020** |
| Quant. IgG mg/dL | 1,760 (1,660; 1,780) | 2,025 (1,595; 2,328) | 2,130 (1,825; 2,475) | 0.436 |
| Bile acids in µmol/L | 4.70 (3.60; 14.4) | 6.50 (3.55; 11.9) | 13.6 (8.50; 24.8) | 0.118 |
| **Liver disease severity** | |  |  |  |
| LSM in kPa | 8.70 (6.80; 11.6) | 14.1 (11.6; 18.6) | 33.7 (29.0; 47.0) | **<0.001** |
| CAP in dB/m | 222 (182; 242) | 196 (180; 288) | 204 (198; 216) | 0.938 |
| ELF test | 9.28 (8.39; 10.3) | 10.5 (9.68; 11.8) | 12.1 (11.5; 12.6) | **0.019** |
| APRI score | 0.51 (0.40; 0.81) | 1.15 (0.71; 1.73) | 1.52 (1.19; 1.70) | **<0.001** |
| FIB-4 score | 1.21 (0.80; 1.60) | 3.54 (2.25; 4.12) | 4.00 (3.06; 5.12) | **<0.001** |
| VITRO score | 0.76 (0.73; 1.15) | 2.15 (1.60; 3.07) | 3.63 (2.76; 4.20) | **0.002** |
| MELD UNOS | 7.00 (6.00; 8.00) | 9.00 (7.00; 10.2) | 11.0 (8.00; 15.5) | **0.008** |
| Ammonia in µmol/L | 21.4 (20.5; 23.3) | 27.7 (25.0; 37.2) | 40.8 (33.2; 45.7) | 0.133 |

**Supplementary Table 1: Application of the Baveno VII criteria for non-invasive diagnosis of portal hypertension in the CHD and CHB cohort.** The stratification was well supported by increasing APRI, FIB-4, and MELD scores. Among CHD patients, von Willebrand antigen increased according to CSPH-probability. Bile acids and markers of systemic inflammation (IL6, PCT) increased with portal hypertension, although not significantly. Rule-in CSPH CHB patients had a high BMI as a co-factor for liver disease severity.
