# Supplementary material for: Chronic hepatitis D in Austria: high burden of cirrhosis and portal hypertension, IL28B C/C as unfavorable factor, increased systemic inflammation in decompensation
Source: Wien Klin Wochenschr. 2026 Feb 5;138(15-16):483–94. doi: 10.1007/s00508-026-02700-4 (PMC13493468; doi:10.1007/s00508-026-02700-4)
Supplement: Supplementary file 2 [file 508_2026_2700_MOESM2_ESM.docx]

|  | ***Decompensation at characterization*** | | |
| --- | --- | --- | --- |
|  | *Compensated (n=72)* | *Decompensated (n=12)* | *pval* |
| Male sex | 39 (54.2%) | 10 (83.3%) | 0.114 |
| BMI | 25.0 (21.9; 28.1) | 26.0 (21.1; 33.5) | 0.580 |
| Daily alcohol intake | 6 (8.3%) | 3 (25.0%) | 0.115 |
| **Viral load** |  |  |  |
| HDV RNA copies/mL | 1.15E+04 (8.75E+01; 2.70E+05) | 1.08E+05 (1.85E+03; 2.65E+05) | 0.870 |
| HBV DNA IU/mL | 1.00E+01 (0.00E+00; 3.30E+01) | 1.00E+01 (0.00E+00; 9.50E+01) | 0.371 |
| HBs antigen IU/mL | 3643 (977; 12594) | 444 (359; 7868) | 0.431 |
| **Lab. Parameters** |  |  |  |
| Platelets in G/L | 164.0 (100.0: 226.0) | 120.0 (59.8; 146.0) | 0.064 |
| AST in U/L | 38.5 (27.8; 56.0) | 50.0 (43.0; 97.0) | **0.024** |
| ALT in U/L | 46.5 (24.2; 88.2) | 71.0 (42.8; 98.5) | 0.254 |
| Bilirubin in mg/dL | 0.62 (0.44; 0.85) | 1.37 (0.87; 2.00) | **0.002** |
| Albumin in mg/dL | 43.9 (40.2; 56.2) | 34.9 (32.3; 39.4) | **0.002** |
| Alk. Phos. in U/L | 78.0 (64.0; 99.0) | 94.0 (84.0; 140.0) | **0.040** |
| Creatinin in mg/dL | 0.78 (0.66; 0.88) | 0.84 (0.79; 0.87) | 0.174 |
| Sodium in mmol/L | 140 (138; 141) | 137 (137; 138) | 0.174 |
| INR | 1.10 (1.00; 1.22) | 1.50 (1.22; 1.70) | **<0.001** |
| vWF antigen percent | 194 (134; 268) | 400 (298; 420) | **0.001** |
| quant. IgG in mg/dL | 1760 (1580; 2130) | 1675 (1558; 1830) | 0.569 |
| Bile acids in µmol/L | 6.35 (3.32; 11.4) | 42.7 (25.6; 87.8) | **0.006** |
| Ammonia in µmol/L | 25.0 (21.1; 33.9) | 77.8 (55.5; 99.1) | **0.025** |
| **Inflammation** |  |  |  |
| CRP in mg/dL | 0.17 (0.10; 0.33) | 0.81 (0.17; 1.20) | 0.061 |
| IL 6 in pg/dL | 3.67 (2.04; 4.45) | 17.5 (13.8; 21.5) | **0.008** |
| PCT in ng/dL | 0.08 (0.04; 0.12) | 0.08 (0.4; 0.16) | 0.740 |
| **Liver disease severity** |  |  |  |
| LSM in kPa | 11.6 (7.0; 16.5) | 17.1 (8.0; 42.5) | **0.006** |
| CAP in dB/m | 213 (182; 263) | 284 (243; 334) | 0.062 |
| ELF score | 10.4 (9.12; 11.0) | 13.2 (11.7; 13.7) | 0.074 |
| FIB4 score | 1.75 (1.17; 3.70) | 3.80 (2.73; 13.0) | **0.010** |
| APRI score | 0.65 (0.35; 1.30) | 1.36 (0.74; 3.70) | **0.018** |
| MELD-UNOS score | 7 (6; 9) | 13 (9; 14) | **<0.001** |
| VITRO score | 1.37 (0.74; 2.74) | 3.39 (2.72; 10.7) | **0.005** |
| HVPG in mmHg | 10.0 (6.0; 13.0) | 18.0 (12.5; 18.5) | **0.027** |

**Supplementary Table 2: Comparison of compensated and decompensated patients at the time of characterization.** Patients with decompensated advanced chronic liver disease had higher levels of biomarkers of systemic and hepatic inflammation, and higher levels of bile acids.
