# Supplementary material for: Chronic hepatitis D in Austria: high burden of cirrhosis and portal hypertension, IL28B C/C as unfavorable factor, increased systemic inflammation in decompensation
Source: Wien Klin Wochenschr. 2026 Feb 5;138(15-16):483–94. doi: 10.1007/s00508-026-02700-4 (PMC13493468; doi:10.1007/s00508-026-02700-4)
Supplement: Supplementary file 3 [file 508_2026_2700_MOESM3_ESM.docx]

|  | ***Anti-HDV positive patients*** | | |
| --- | --- | --- | --- |
|  | *HDV RNA positive (n=59)* | *HDV RNA negative (n=25)* | *pval* |
| Male sex | 35 (59.3%) | 14 (56.0%) | 0.968 |
| BMI | 23.8 (21.8; 28.7) | 27.0 (21.0; 30.1) | 0.360 |
| Daily alcohol intake | 6 (10.2%) | 3 (12.0%) | 1.000 |
| **Viral load** |  |  |  |
| HDV RNA copies/mL | 1.25E+05 (8.33E+03; 4.25E+05) | 0.00E+00 (0.00E+00; 0.00E+00) | **<0.001** |
| HBV DNA IU/mL | 1.00E+01 (0.00E+00; 2,35E+01) | 2.5E+01 (0.00E+00; 1.27E+02) | 0.056 |
| HBs antigen IU/mL | 7514 (2090; 14079) | 42.3 (0; 1429) | **<0.001** |
| **Lab. Parameters** |  |  |  |
| Platelets in G/L | 134 (91.5; 204) | 193 (140; 253) | **0.035** |
| AST in U/L | 51.0 (39.0; 68.0) | 25.0 (19.0, 30.0) | **<0.001** |
| ALT in U/L | 66.0 (42.0; 107.0) | 20.0 (19.0; 30.0) | **<0.001** |
| Bilirubin in mg/dL | 0.66 (0.50; 1.10) | 0.62 (0.34; 0:81) | 0.146 |
| Albumin in mg/dL | 42.7 (38.1; 44.5) | 45.6 (43.4; 47.6) | **0.008** |
| Alk. Phos. in U/L | 83.0 (71.0; 120.0) | 67.0 (55.5; 80.0) | **0.007** |
| Creatinin in mg/dL | 0.79 (0.65; 0.86) | 0.80 (0.68; 0.91) | 0.296 |
| Sodium in mmol/L | 139 (137; 140) | 141 (138; 142) | **0.044** |
| INR | 1.20 (1.0; 1.30) | 1.10 (1.0; 1.20) | 0.113 |
| vWF antigen percent | 253 (170; 326) | 126 (120; 246) | 0.066 |
| quant. IgG in mg/dL | 1780 (1640; 2145) | 1570 (1160; 1650) | 0.061 |
| Bile acids in µmol/L | 6.60 (3.62; 14.2) | 4.65 (1.95; 74.9) | 0.575 |
| Ammonia in µmol/L | 27.0 (21.5; 39.1) | 22.0 (18.0; 33.4) | 0.533 |
| **Inflammation** |  |  |  |
| CRP in mg/dL | 0.29 (0.11; 0.47) | 0.12 (0.06; 0.27) | 0.091 |
| IL 6 in pg/dL | 3.76 (2.65; 4.80) | 8.51 (2.88; 28.4) | 0.416 |
| PCT in ng/dL | 0.08 (0.04; 0.14) | 0.06 (0.03; 0.10) | 0.567 |
| **Liver disease severity** |  |  |  |
| LSM in kPa | 13.1 (8.6; 18.9) | 5.1 (4.8; 7.1) | **0.001** |
| CAP in dB/m | 203 (182; 259) | 248 (216; 279) | 0.236 |
| ELF score | 10.5 (9.45; 11.6) | 11.2 (9.79; 12.7) | 0.835 |
| FIB4 score | 2.40 (1.27; 3.98) | 1.44 (0.96; 2.15) | 0.092 |
| APRI score | 0.83 (0.54; 1.70) | 0.28 (0.24; 0.60) | **<0.001** |
| MELD-UNOS score | 8 (7; 10) | 7 (6; 8) | 0.103 |
| VITRO score | 1.82 (0.86; 3.51) | 0.87 (0.54; 2.26) | 0.106 |
| HVPG in mmHg | 10.5 (6.0; 17.0) | 8.5 (7.0; 11.0) | 0.693 |

**Supplementary Table 3: Comparison of HDV RNA positive and HDV RNA negative patients with anti-HDV antibodies.**
