# Supplementary material for: Chronic hepatitis D in Austria: high burden of cirrhosis and portal hypertension, IL28B C/C as unfavorable factor, increased systemic inflammation in decompensation
Source: Wien Klin Wochenschr. 2026 Feb 5;138(15-16):483–94. doi: 10.1007/s00508-026-02700-4 (PMC13493468; doi:10.1007/s00508-026-02700-4)
Supplement: Supplementary file 4 [file 508_2026_2700_MOESM4_ESM.docx]

|  | ***Decompensation at characterization*** | | | | | |
| --- | --- | --- | --- | --- | --- | --- |
|  | *HDV viremic* | |  | *Anti-HDV positive, non-viremic* | |  |
|  | *Compensated (n=50)* | *Decompensated (n=9)* | *pval* | *Compensated, HDV non-viremic (n=22)* | *Decompensated, HDV non-viremic (n=3)* | *pval* |
| Male sex | 28 (56.0%) | 7 (77.7%) | 0.287 | 11 (50.0%) | 3 (100.0%) | 0.230 |
| BMI | 24.3 (22.1; 28.6) | 21.8 (20.5; 26.0) | 0.236 | 25.4 (20.9; 27.9) | 36.0 (35.1; 38.0) | **0.017** |
| Daily alcohol intake | 4 (8.0%) | 2 (22.2%) | 0.224 | 2 (9.1%) | 1 (33.3%) | 0.330 |
| **Viral load** |  |  |  |  |  |  |
| HDV RNA copies/mL | 1.18E+05 (8.30E+03; 4.45E+05) | 1.50E+05 (8.71E+04; 3.58E+05) | 0.693 | 0.00E+00 (0.00E+00; 0.00E+00) | 0.00E+00 (0.00E+00; 0.00E+00) | 0.554 |
| HBV DNA IU/mL | 0.00E+00 (0.00E+00; 0.00E+00; 2.00E+01) | 1.0E+1 (5.0E+00; 6.35E+01) | 0.257 | 2.50E+01 (5.00E+00; 1.20E+02) | 3.75E+04 (1.88E+04; 5.63E+04) | 0.763 |
| HBs antigen IU/mL | 7782 (2637; 14263) | 2090 (444; 13647) | 0.509 | 42 (0; 1560) | 161 (80; 242) | 0.629 |
| **Lab. Parameters** |  |  |  |  |  |  |
| Platelets in G/L | 136 (90.8; 208) | 124 (109; 165) | 0.658 | 208 (155; 265) | 50 (36; 95) | **0.015** |
| AST in U/L | 51.5 (36.8; 68.0) | 47.0 (43.0; 87.0) | 0.490 | 23.0 (17.8; 28.2) | 106.0 (67.0; 781) | **0.025** |
| ALT in U/L | 64.5 (42.0; 118) | 69.0 (45.0; 98.0) | 0.776 | 20.0 (18.8; 24.0) | 78.0 (48.5; 489) | 0.131 |
| Bilirubin in mg/dL | 0.65 (0.50; 0.95) | 1.15 (0.64; 1.43) | 0.105 | 0.46 (0.33; 0.74) | 13.8 (8.08; 13.9) | **0.006** |
| Albumin in mg/dL | 43.1 (39.4; 44.5) | 36.5 (33.8; 41.7) | 0.103 | 46.2 (44.1; 47.7) | 30.9 (30.6; 34.5) | **0.012** |
| Alk. Phos. in U/L | 81.0 (67.0; 118) | 95.0 (81.5; 135) | 0.402 | 66.0 (55.0; 77.2) | 94.0 (88.5; 306) | **0.010** |
| Creatinin in mg/dL | 0.76 (0.65; 0.88) | 0.83 (0.77; 0.84) | 0.715 | 0.79 (0.66; 0.87) | 1.90 (1.40; 2.83) | **0.021** |
| Sodium in mmol/L | 140 (138; 141) | 137 (137; 138) | **0.018** | 141 (139; 142) | 137 (131; 140) | 0.186 |
| INR | 1.13 (1.00; 1.30) | 1.30 (1.20; 1.65) | **0.014** | 1.06 (1.00; 1.10) | 2.00 (1.70; 2.05) | **0.005** |
| vWF antigen percent | 245 (165; 316) | 358 (303; 405) | **0.028** | 121 (118; 132) | 420 (350; 420) | **0.014** |
| quant. IgG in mg/dL | 1820 (1700; 2190) | 1570 (1545; 1675) | 0.162 | 1365 (1040; 1590) | 1980 (1980; 1980) | 0.157 |
| Bile acids in µmol/L | 6.50 (3.60; 12.7) | 26.0 (19.8; 34.8) | 0.072 | 2.00 (1.77; 3.38) | 108 (103; 114) | 0.064 |
| Ammonia in µmol/L | 26.1 (21.5; 35.6) | 66.7 (45.8; 83.1) | 0.089 | 20.0 (16.7; 24.9) | 218 (218; 218) | 0.157 |
| **Inflammation** |  |  |  |  |  |  |
| CRP in mg/dL | 0.29 (0.11; 0.42) | 0.48 (0.19; 1.14) | 0.191 | 0.11 (0.05; 0.16) | 1.36 (0.71; 10.7) | 0.184 |
| IL 6 in pg/dL | 3.71 (2.40; 4.47) | 15.3 (9.04; 18.8) | 0.144 | 3.34 (1.50; 3.71) | 19.6 (16.5; 37.2) | 0.177 |
| PCT in ng/dL | 0.08 (0.04; 0.13) | 0.08 (0.06; 0.12) | 0.928 | 0.06 (0.04; 0.08) | 3.75 (1.89; 5.61) | 0.564 |
| **Liver disease severity** |  |  |  |  |  |  |
| LSM in kPa | 13.2 (8.8; 18.2) | 11.6 (7.6; 27.1) | 0.928 | 5.00 (4.65; 6.15) | 75.0 (75.0; 75.0) | 0.110 |
| CAP in dB/m | 198 (181; 241) | 255 (230; 284) | 0.171 | 245 (213; 264) | 398 (398; 398) | 0.117 |
| ELF score | 10.5 (9.36; 11.2) | 11.7 (10.9; 12.4) | 0.304 | 8.33 (8.33; 8.33) | 14.2 (14.2; 14.2) | 0.317 |
| FIB4 score | 2.07 (1.26; 3.89) | 3.05 (2.42; 4.46) | 0.370 | 1.41 (0.78; 1.81) | 22.2 (14.1; 22.3) | **0.006** |
| APRI score | 0.83 (0.53; 1.66) | 0.81 (0.71; 1.71) | 0.615 | 0.27 (0.23; 0.36) | 9.64 (5.38; 15.2) | **0.006** |
| MELD-UNOS score | 8.0 (6.75; 10.0) | 9.5 (9.0; 12.2) | **0.045** | 7.0 (6.0; 7.25) | 30.0 (22.0; 33.5) | **0.004** |
| VITRO score | 1.65 (0.80; 3.32) | 2.92 (2.44; 6.48) | 0.124 | 0.60 (0.50; 0.92) | 5.62 (4.31; 12.4) | **0.014** |
| HVPG in mmHg | 10.0 (5.5; 13.5) | 18.0 (18.0; 19.0) | **0.021** | 8.50 (7.75; 9.25) | 11.0 (9.0; 13.0) | 0.683 |

**Supplementary Table 4: Comparing patients with compensated and decompensated liver disease with regards to HDV RNA positivity.**
