# Supplementary material for: Chronic hepatitis D in Austria: high burden of cirrhosis and portal hypertension, IL28B C/C as unfavorable factor, increased systemic inflammation in decompensation
Source: Wien Klin Wochenschr. 2026 Feb 5;138(15-16):483–94. doi: 10.1007/s00508-026-02700-4 (PMC13493468; doi:10.1007/s00508-026-02700-4)
Supplement: Supplementary file 5 [file 508_2026_2700_MOESM5_ESM.docx]

|  | ***IL28B status*** | |  |
| --- | --- | --- | --- |
|  | *C/T, T/T (n=19)* | *C/C (n=17)* |  |
| Male sex | 11 (57.9%) | 10 (58.8%) | 1.000 |
| BMI | 25.9 (23.6; 30.0) | 22.1 (20.7; 26.5) | **0.034** |
| Daily alcohol intake | 2 (10.5%) | 2 (11.8%) | 1.000 |
| **Viral load** |  |  |  |
| HDV RNA | 1.6E+05 (1.2E+04; 3.9E+05) | 2.45E+5 (1.06E+05; 1.18E+06) | 0.191 |
| HBV DNA | 0 (0; 2.6E+01) | 0 (0; 1.3E+01) | 0.970 |
| HBs antigen | 2,642 (1,032; 14,079) | 6,200 (1,590; 12,192) | 0.955 |
| **Lab. parameters** |  |  |  |
| Platelets in G/L | 166 (110; 241) | 98 (77; 134) | **0.019** |
| AST in U/L | 54.0 (42.5; 83.5) | 56.0 (52.0; 87.0) | 0.754 |
| ALT in U/L | 73.0 (43.0; 125) | 73.0 (44.0; 104.0) | 0.912 |
| Bilirubin in mg/dL | 0.62 (0.48; 1.02) | 0.95 (0.52; 1.36) | 0.214 |
| Albumin in mg/dL | 42.6 (41.0; 44.2) | 37.3 (33.7; 39.9) | **0.010** |
| Alk. Phos. In U/L | 90.0 (73.5; 147.0) | 114 (82.0; 139.0) | 0.679 |
| Creatinin in mg/dL | 0.79 (0.70; 0.97) | 0.76 (0.62; 0.88) | 0.267 |
| Sodium in mmol/L | 139 (137; 140) | 139 (137; 141) | 1.000 |
| INR | 1.1 (1.0; 1.2) | 1.3 (1.2; 1.6) | **0.002** |
| quant. IgG in mg/dL | 1,780 (1,570; 2090) | 2,135 (1,700; 2385) | 0.336 |
| Bile acids in µmol/L | 7.00 (4.4; 10.8) | 13.6 (6.60; 36.4) | 0.077 |
| vWF antigen percent | 250 (202; 320) | 262 (166; 332) | 0.839 |
| Ammonia in µmol/L | 20.9 (16.4; 29.1) | 37.7 (28.9; 53.5) | **0.013** |
| **Inflammation** |  |  |  |
| CRP in mg/dL | 0.28 (0.10; 0.44) | 0.13 (0.11; 0.48) | 0.733 |
| IL6 in pg/mL | 3.91 (2.78; 4.45) | 4.53 (3.67; 9.23) | 0.233 |
| PCT in ng/mL | 0.08 (0.05; 0.11) | 0.13 (0.08; 0.16) | 0.229 |
| **Liver disease severity** |  |  |  |
| Ascites | 1 (5.3%) | 3 (17.6%) | 0.315 |
| Hepatic encephalopathy | 2 (10.5%) | 4 (23.5%) | 0.383 |
| LSM in kPa | 13.6 (9.7; 19.2) | 16.6 (9.50; 25.4) | 0.613 |
| F0-1 | 1 (5.6%) | 1 (6.7%) | 1.000 |
| F2 | 2 (11.1%) | 3 (20.0%) |  |
| F3 | 3 (16.7%) | 1 (6.7%) |  |
| F4 | 12 (66.7%) | 10 (66.7%) |  |
| CAP in dB/m | 216 (192; 291) | 189 (178; 212) | 0.095 |
| Biopsy F4 | 5 (35.7%) | 9 (81.8%) | 0.051 |
| ELF score | 10.4 (9.7; 11.3) | 10.9 (10.5; 11.8) | 0.441 |
| VITRO score | 1.75 (0.78; 2.71) | 2.70 (1.79; 4.31) | 0.441 |
| APRI score | 0.82 (0.57; 1.68) | 1.36 (0.80; 2.98) | 0.156 |
| FIB4 score | 2.10 (1.32; 3.66) | 3.76 (2.84; 5.94) | 0.120 |
| MELD | 8.0 (6.0; 9.0) | 10.5 (8.75; 12.2) | **0.013** |
| HVPG in mmHg | 6.5 (4.0; 10.0) | 17.0 (10.5; 19.0) | **0.005** |

**Supplementary Table 5: CHD patients’ characteristics stratified by IL28B status**. Patients with the “favorable” C/C genotype exhibited signs of more severe chronic liver disease compared to C/G or G/G. Patients with a C/C single nucleotide polymorphism had higher LSM (not significant), higher HVPG and MELD score, higher blood levels of ammonia, higher INR, lower albumin and platelets.
