# Supplementary material for: Chronic hepatitis D in Austria: high burden of cirrhosis and portal hypertension, IL28B C/C as unfavorable factor, increased systemic inflammation in decompensation
Source: Wien Klin Wochenschr. 2026 Feb 5;138(15-16):483–94. doi: 10.1007/s00508-026-02700-4 (PMC13493468; doi:10.1007/s00508-026-02700-4)
Supplement: Supplementary file 6 [file 508_2026_2700_MOESM6_ESM.docx]

|  | ***PNPLA3 status*** | |  |
| --- | --- | --- | --- |
|  | *C/C (n=14)* | *G/G, C/G (n=25)* |  |
| Male sex | 10 (71.4%) | 16 (64.0%) | 0.733 |
| BMI | 25.1 (22.4; 29.6) | 23.8 (20.8; 27.6) | 0.296 |
| Daily alcohol intake | 3 (21.4%) | 1 (4.0%) | 0.123 |
| **Viral load** |  |  |  |
| HDV RNA | 2.6E+05 (1.0E+05; 7.1E+05) | 1.8E+05 (1.1E+04; 5.4E+05) | 0.525 |
| HBV DNA | 0 (0; 2.6E+01) | 1.00E+01 (0; 2.7E+01) | 0.345 |
| HBs antigen | 8,033 (1,678; 20,755) | 4,673 (1,090; 13,680) | 0.627 |
| **Lab. parameters** |  |  |  |
| Platelets in G/L | 118 (86.5; 193) | 112 (84.0; 177) | 0.988 |
| AST in U/L | 51.0 (41.8; 64.5) | 55.5 (42.8; 88.5) | 0.440 |
| ALT in U/L | 66.0 (44.2; 96.2) | 78.0 (47.0; 125) | 0.438 |
| Bilirubin in mg/dL | 0.80 (0.62; 1.12) | 0.80 (0.50; 1.35) | 0.782 |
| Albumin in mg/dL | 38.3 (34.8; 42.4) | 41.7 (37.4; 44.0) | 0.438 |
| Alk. Phos. In U/L | 118 (83.0; 152) | 85.0 (79.0; 136) | 0.364 |
| Creatinin in mg/dL | 0.86 (0.77; 1.01) | 0.77 (0.69; 0.80) | 0.051 |
| Sodium in mmol/L | 139 (137; 141) | 139 (137; 140) | 0.883 |
| INR | 1.30 (1.02; 1.30) | 1.20 (1.00; 1.30) | 0.471 |
| quant. IgG in mg/dL | 1,660 (1,545; 2,220) | 1,780 (1,760; 2,380) | 0.791 |
| Bile acids in µmol/L | 14.4 (7.85; 25.8) | 6.60 (4.80; 12.6) | 0.082 |
| vWF antigen percent | 320 (268; 383) | 220 (159; 279) | **0.027** |
| Ammonia in µmol/L | 26.2 (20.3; 38.4) | 37.7 (26.8; 55.1) | 0.248 |
| **Inflammation** |  |  |  |
| CRP in mg/dL | 0.32 (0.25; 0.53) | 0.12 (0.09; 0.29) | **0.006** |
| IL 6 in pg/mL | 4.18 (2.79; 9.75) | 4.10 (3.67; 4.53) | 0.847 |
| PCT in ng/mL | 0.11 (0.08; 0.14) | 0.08 (0.05; 0.14) | 0.529 |
| **Liver disease severity** |  |  |  |
| Ascites | 2 (14.3%) | 4 (16.0%) | 1.000 |
| Hepatic encephalopathy | 3 (21.4%) | 5 (20.0%) | 1.000 |
| LSM in kPa | 16.6 (13.2; 28.4) | 10.5 (8.10; 20.2) | 0.063 |
| F0-1 | 0 (0%) | 2 (8.7%) | 0.161 |
| F2 | 1 (7.7%) | 6 (26.1%) |  |
| F3 | 1 (7.7%) | 5 (21.7%) |  |
| F4 | 11 (84.6%) | 10 (43.5%) |  |
| CAP in dB/m | 204 (192; 246) | 191 (178; 229) | 0.620 |
| Biopsy F4 | 8 (72.7%) | 6 (46.2%) | 0.240 |
| ELF score | 11.8 (10.7; 12.3) | 10.7 (9.62; 10.9) | 0.064 |
| VITRO score | 2.79 (2.46; 3.32) | 1.82 (1.35; 4.26) | 0.468 |
| APRI score | 1.31 (0.66; 1.63) | 1.11 (0.72; 2.50) | 0.650 |
| FIB4 score | 2.99 (1.93; 4.72) | 3.54 (1.54; 4.34) | 0.952 |
| MELD | 9.00 (7.0; 11.0) | 8.00 (6.00; 11.0) | 0.469 |
| HVPG in mmHg | 11.0 (10.0; 14.5) | 10.0 (5.00; 18.0) | 0.576 |

**Supplementary Table 6: Characteristics of CHD patients stratified by PNPLA3 status.** “Unfavorable” PNPLA3 genotypes G/G and G/C did not affect steatosis or liver stiffness (CAP and LSM).
